# Neutrophil count and urinary glucose as early predictors of gestational diabetes mellitus in nulliparous women of advanced maternal age: a retrospective cohort study

**DOI:** 10.3389/fendo.2026.1791755

**Published:** 2026-04-10

**Authors:** Kai Sun Zhao, Jie Cheng Bi, Ni Bei, Jian Chun Huang, Mei Rong He, Chun Lan Yuan

**Affiliations:** 1 Department of Obstetrics, The Third Affiliated Hospital of Guangxi Medical University, the Second Nanning People’s Hospital, Nanning, Guangxi Zhuang Autonomous Region, China; 2 China-ASEAN Medical Research Center, China Hospital Development Institute, Shanghai Jiao Tong University, Shanghai, China

**Keywords:** advanced maternal age, assisted reproductive technology, first trimester, gestational diabetes mellitus, glycosuria, neutrophils, prediction

## Abstract

**Background:**

Gestational diabetes mellitus (GDM) poses significant risks, particularly for nulliparous women of advanced maternal age (AMA ≥35 years), a growing demographic due to delayed childbirth. However, effective early-pregnancy prediction tools tailored for this high-risk subgroup are lacking. This study aimed to identify readily available early biomarkers for GDM risk stratification in AMA nulliparous women.

**Methods:**

This retrospective cohort study analyzed 354 AMA nulliparous women (140 GDM, 214 non-GDM). GDM was diagnosed by a 75g oral glucose tolerance test at 24–28 weeks using IADPSG criteria (fasting ≥5.1 mmol/L, 1-h ≥10.0 mmol/L, 2-h ≥8.5 mmol/L). Clinical and laboratory data at 10–13 gestational weeks were compared. Multivariate logistic regression identified independent risk factors.

**Results:**

The GDM group had significantly higher body mass index (BMI), fasting blood glucose (FBG), white blood cell (WBC) count, neutrophil count (NEU), and urinary glucose (U-GLU) positivity (P< 0.05). Assisted reproductive technology (ART) use did not differ significantly in univariate analysis (P = 0.083). Multivariable analysis identified U-GLU positivity (adjusted odds ratio [aOR] = 7.91; 95% CI: 2.67-23.46), elevated FBG (aOR = 2.23 per mmol/L; 95% CI: 1.13-4.38), elevated NEU (aOR = 1.21 per 10^9^/L; 95% CI: 1.05-1.40), elevated WBC (aOR = 1.15 per 10^9^/L; 95% CI: 1.01-1.30), and ART use (aOR = 1.63; 95% CI: 1.02-2.59) as independent risk factors for GDM. The multivariable model achieved an AUC of 0.70 (95% CI 0.65 - 0.76), with sensitivity of 57.1% and specificity of 76.2% at the optimal cutoff.

**Conclusions:**

In this single-center retrospective cohort of AMA nulliparous women, early-pregnancy urinary glucose positivity, elevated fasting blood glucose, neutrophilia, leukocytosis, and the use of assisted reproductive technology were independently associated with an increased risk of GDM, with urinary glucose showing the strongest association. These findings suggest that a tiered screening strategy incorporating these readily available biomarkers might be explored for early risk stratification between 10–13 weeks’ gestation. Their clinical utility requires prospective validation in larger, multicenter cohorts.

## Introduction

Gestational diabetes mellitus (GDM) is a common metabolic complication affecting approximately 15–20% of pregnancies globally, according to recent estimates by the International Diabetes Federation (IDF) (1, 2). The prevalence varies significantly across regions, reaching up to 25% in parts of Southeast Asia and the Middle East, while remaining around 5–10% in Western countries. In China, national studies report a rising trend, with prevalence rates between 8.6% and 16.7% (3, 4), reflecting growing public health concerns.

In recent years, trends toward delayed marriage and childbirth (5), coupled with the growing use of assisted reproductive technology (ART) (6, 7), have increased the proportion of women experiencing their first pregnancy at an advanced maternal age (AMA; ≥35 years at delivery). This demographic shift has attracted substantial public health attention, as AMA nulliparous women represent a high-risk group; advanced maternal age is a well-established independent risk factor for GDM (1, 5, 8), with this population facing approximately a 20% increased risk. Such elevated risk is strongly associated with adverse maternal and neonatal outcomes (9–11). Despite the well-established link between AMA nulliparity and GDM, a critical knowledge gap remains: current clinical guidelines primarily emphasize conventional risk factors such as obesity and family history of diabetes (1, 8, 12), but lack a comprehensive risk stratification system that incorporates early-pregnancy inflammatory and metabolic biomarkers specifically for this high-risk subgroup. Early identification of women at greatest risk could enable targeted interventions and potentially improve outcomes.To address this gap, we conducted a retrospective cohort study to systematically investigate the risk profile for GDM—including white blood cell (WBC) count, neutrophil (NEU) count, and glycosuria—specifically among AMA nulliparous women. The aim is to provide evidence-based insights to optimize early risk stratification and ultimately improve long-term maternal and neonatal health outcomes.

## Patients and methods

### Ethical considerations and study design

This retrospective cohort study was designed in accordance with the PICOS framework: Participants were nulliparous women of advanced maternal age (≥35 years); Intervention/Exposure referred to early-pregnancy biomarkers, including NEU, WBC, urinary glucose (U-GLU), fasting blood glucose (FGB), and use of assisted reproductive technology; Comparison was made between those who developed GDM and those who did not; the primary Outcome was the diagnosis of GDM at 24–28 weeks’ gestation; and the overall Study design was a single-center retrospective cohort analysis.

This study is a retrospective observational cohort study. In accordance with the Declaration of Helsinki (World Medical Association Inc., 2009), this study required ethical review and approval. Ethical approval was obtained from the Ethics Committee of Nanning Second People’s Hospital, following institutional guidelines and national regulations. Due to its retrospective nature, the study was approved by the Ethics Committee of Nanning Second People’s Hospital with a waiver of informed consent.

### Laboratory measurements

FBG was measured using standard automated analyzers in the central laboratory of our institution following an overnight fast of at least 8 hours. WBC, NEU, hemoglobin (HB), and other hematological parameters were measured using routine automated hematology analyzers at the same laboratory. All blood samples were collected between 10 and 13 weeks of gestation and processed according to standard clinical protocols.Urinalysis was performed on fasting morning urine samples collected on the same day as blood sampling. All urine samples were tested within 1 hour of collection using the DIRUI MUS-9600 automated urinalysis system (DIRUI Medical Technology Co., Ltd., Changchun, China; Medical Device Registration No. Jixiezhuzhun 20202220162). Dipstick results were read automatically and confirmed manually by trained laboratory technicians. U-GLU positivity was defined as ≥1+ on semi-quantitative dipstick testing. All laboratory tests were performed blinded to patient outcomes.

### Study population and data collection

The inclusion criteria were as follows: singleton pregnancy in advanced maternal age nulliparous women; early pregnancy registration (≤13 weeks of gestation) with regular prenatal care; completion of a standard 75g oral glucose tolerance test (OGTT) between 24 and 28 weeks of gestation; and availability of complete clinical and laboratory data, including demographic characteristics, biomarkers, and pregnancy outcomes.

Exclusion criteria included multiparity (history of previous live birth), maternal age<35 years, multiple gestations, pre-existing medical conditions such as diabetes mellitus, chronic kidney disease, autoimmune disorders, or endocrine diseases affecting glucose metabolism (e.g., hyperthyroidism, hypothyroidism, Cushing’s syndrome), missing critical data (including OGTT results, FBG, WBC, NEU, U-GLU, or ART records), and use of corticosteroid therapy during pregnancy.

**Figure 1:**
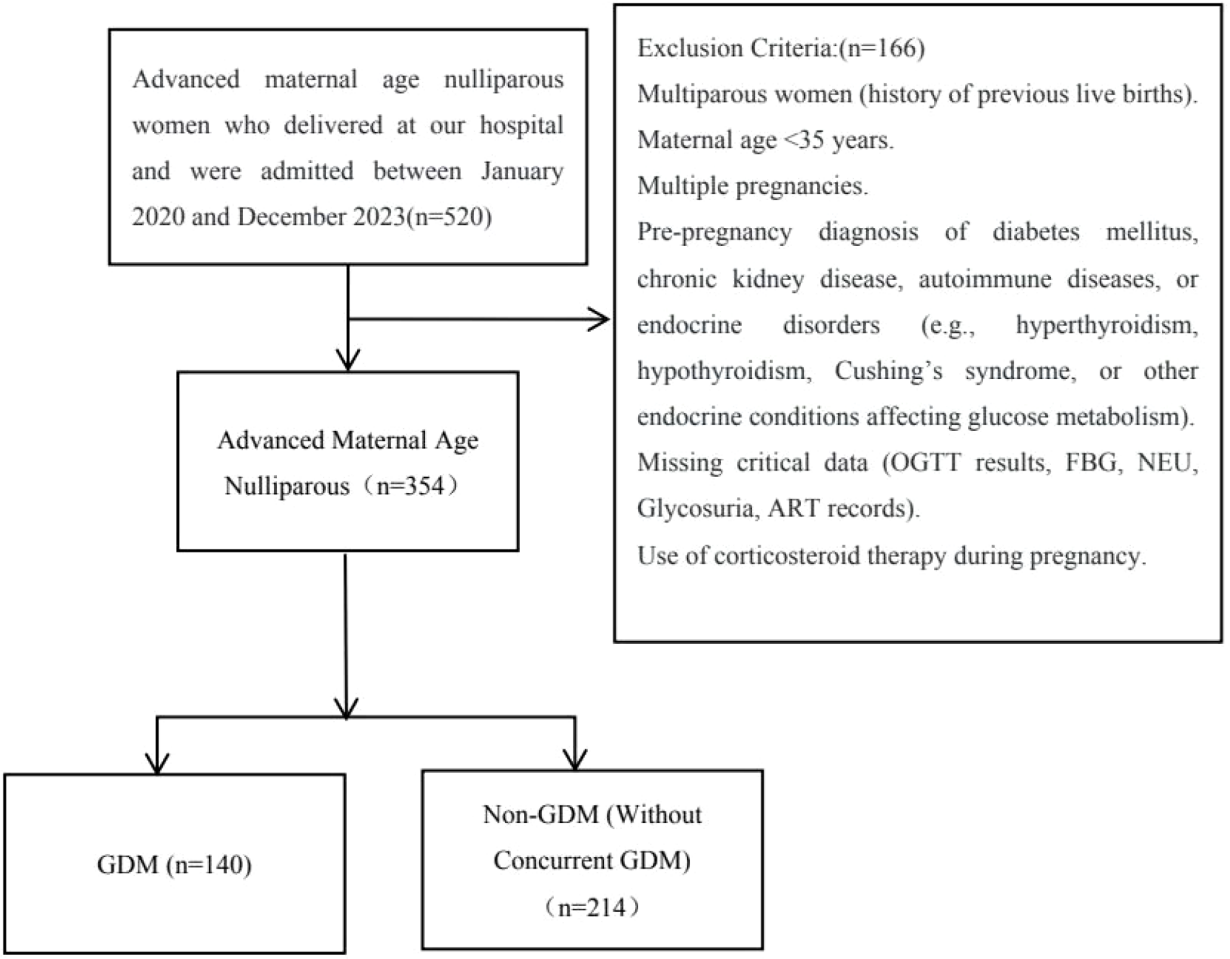
Flowchart of patient screening process.

We retrospectively collected data from 520 nulliparous women of advanced maternal age who delivered at our institution between January 2020 and December 2023. After reviewing medical records, 166 women were excluded because they had not established early prenatal care or undergone laboratory testing at our hospital during the first trimester (10–13 weeks of gestation); consequently, their complete first-trimester clinical and biomarker data were unavailable. The remaining 354 women with complete data constituted the final study cohort. All ART cases (n = 152) were included in the unified analysis, comprising 5 cases of intrauterine insemination (IUI) and 147 cases of *in vitro* fertilization–embryo transfer (IVF-ET). Due to the small sample size of the IUI subgroup, stratified analysis by ART type was not performed. Blood and urine biomarkers were assessed between 10 and 13 weeks of gestation. The case selection flowchart is presented in **Figure 1**.

### Diagnosis of gestational diabetes mellitus

GDM was diagnosed based on the criteria recommended by the Chinese Society of Obstetrics and Gynecology (2022 edition) and International Association of Diabetes and Pregnancy Study Groups (IADPSG) (13, 14), which follow the IADPSG thresholds using a 75-g OGTT performed between 24 and 28 weeks of gestation. A diagnosis of GDM was established if any one of the following plasma glucose values was met or exceeded: fasting glucose ≥5.1 mmol/L, 1-hour post-load glucose ≥10.0 mmol/L, or 2-hour post-load glucose ≥8.5 mmol/L.

Grouping method:

140 cases with GDM, 214 cases Non-GDM.

Observation indicators:

1. Baseline data: age, gravidity, body mass index (BMI), age at menarche, etc.
2. Laboratory indicators: early pregnancy FBG, WBC, NEU, HB, U-GLU, etc.
3. Clinical-related variables: ART.

### Statistical analysis

Statistical analyses were performed using SPSS 29.0, Zstats 1.0 (www.zstats.net), the Storm platform (www.medsta.cn/software), and R version 4.3.3. Normality of continuous variables was assessed using the Kolmogorov–Smirnov test. Normally or approximately normally distributed continuous data were expressed as mean ± standard deviation ( $\bar{x}$ ± s), and comparisons between groups were conducted using the t-test. A P-value< 0.05 was considered statistically significant. Non-normally distributed continuous data were expressed as medians (interquartile range), and group comparisons were performed with the Mann-Whitney U test; P< 0.05 was considered statistically significant. Categorical data were expressed as percentages (%) and analyzed using the χ² test; when expected counts were less than 5, Fisher’s exact test was used. A P<0.05 indicated statistically significant differences. Binary logistic regression analysis identified independent risk factors for GDM. Univariate analysis was first performed; to avoid missing clinically relevant factors, Candidate variables for the multivariable logistic regression model were selected based on clinical relevance and results of univariate analysis (P< 0.10). Odds ratios (OR) with 95% confidence intervals (95% CI) were calculated. The predictive performance of the multivariable model was evaluated using several metrics. Discrimination was assessed by the area under the receiver operating characteristic curve (AUC). Calibration was evaluated using a calibration plot and the Hosmer–Lemeshow goodness-of-fit test, with a non-significant P-value indicating good calibration. At the optimal probability cutoff determined by the Youden index, sensitivity, specificity, positive predictive value (PPV), and negative predictive value (NPV) were calculated based on the study population’s GDM prevalence. Decision curve analysis (DCA) was performed to assess the clinical net benefit of the model across a range of threshold probabilities.To complement the logistic regression results, SHapley Additive exPlanations (SHAP) analysis was applied. SHAP is a model-agnostic framework that quantifies the contribution of each feature to individual predictions, providing a visual and interpretable summary of variable importance. SHAP values were derived from the logistic regression model and visualized in a beeswarm plot, illustrating the direction and magnitude of each predictor’s effect at the individual level.*Post-hoc* power analysis was performed using R software (version 4.3.3) based on the observed effect sizes and sample sizes. For urinary glucose positivity, with proportions of 13.6% in the GDM group and 2.3% in the non-GDM group, the statistical power exceeded 99% at a two-sided alpha level of 0.05, indicating that the sample size was more than sufficient to detect this strong association. For ART use, based on the observed exposure rates (48.6% in GDM vs. 39.3% in non-GDM), the power was approximately 80%, suggesting adequate power to detect this moderate effect. These calculations confirm that the study had sufficient statistical power to identify the main independent risk factors.

## Results

### Comparison of baseline data

Comparison of baseline data between complete and incomplete datasets showed no statistically significant differences in age, gravidity, BMI, or age at menarche (P > 0.05), (**Table 1**).

**Table 1:** Comparison of baseline data between complete and incomplete datasets.

	Incomplete datasets (n=166)	Complete datasets (n=354)	Z	*P*
Age (Year)	37.00 (35.75-39.00)	37.00 (35.000-38.00)	-0.632	0.527
BMI (kg/m²)	22.03 (19.942-24.40)	22.18 (20.28-24.44)	-0.207	0.836
Gravidity	2.00(1.00-3.00)	2.00 (1.00-2.25)	-1.732	0.083
Age at menarche (Year)	13.00 (12.00-13.00)	13.00 (13.00-14.00)	-0.869	0.385

BMI, body mass index.

### Comparison of baseline data between GDM and non-GDM groups

There were no statistically significant differences in age, gravidity, or age at menarche between the GDM and non-GDM groups (P > 0.05). BMI: The median BMI in the GDM group was 22.68 (IQR: 20.57–24.89), while in the non-GDM group it was 21.90 (IQR: 20.03–23.73). The comparison between the two groups showed a statistically significant difference (P = 0.014), (**Table 2**).

**Table 2:** Comparison of baseline data between GDM and non-GDM groups in complete data.

	Non-GDM (n=214)	GDM (n=140)	Z	*P*
Age (Year)	37.00 (35.00-38.00)	37.00 (36.00-38.00)	-1.666	0.096
BMI (kg/m²)	21.90 (20.03-23.73)	22.68 (20.57-24.89)	-2.449	0.014
Gravidity	2.00 (1.00-2.75)	2.00 (1.00-2.25)	-0.189	0.850
Age at menarche (Year)	13.00 (13.00-14.00)	13.00 (12.00-14.00)	-0.558	0.577

BMI, body mass index.

### Comparison of laboratory indicators and clinical variables between GDM and non-GDM groups

Since IUI cases account for only 3.29% of the total ART cases (5/152), subsequent analysis will treat ART as a single variable, with its risk effects mainly reflecting the characteristics of the IVF-ET subgroup. There were statistically significant differences between the two groups in FBG, WBC, NEU, and U-GLU (P< 0.05). No statistically significant differences were observed in HB and ART between the two groups (P > 0.05), (**Table 3**).

**Table 3:** Comparison of laboratory indicators and clinical variables between GDM and non-GDM groups.

	Non-GDM (n=214)	GDM (n=140)	t/z/χ²	P
FBG(mmol/L)	4.63 ± 0.31	4.71 ± 0.43	2.08	0.038
WBC (10^9^/L)	8.00 ± 2.05	9.11 ± 2.41	4.615	<0.001
NEU (10^9^/L)	5.63 (4.51-7.08)	6.50 (5.37-7.84)	-4.425	<0.001
HB (g/L)	122.00 (115.00-129.00)	124 (117.50-131.00)	-1.605	0.109
ART	84.00 (39.25%)	68.00(48.57%)	3.000	0.083
U-GLU	5.00 (2.34%)	19.00 (13.57%)	16.903	<0.001

FBG, fasting blood glucose; WBC, white blood cells; NEU, neutrophils; HB, hemoglobin; ART, assisted reproductive technology, U-GLU, urinary glucose.

### Multivariate logistic regression analysis of risk factors for GDM in advanced maternal age nulliparous women

The final multivariable logistic regression model included ART, U-GLU positivity, FBG, NEU, WBC, and BMI (**Table 4**). After adjusting for these variables, U-GLU positivity (aOR = 7.91, 95% CI: 2.67–23.46, P< 0.001) remained the strongest independent risk factor, followed by elevated FBG (aOR = 2.23, 95% CI: 1.13–4.38, P = 0.020), elevated NEU (aOR = 1.21, 95% CI: 1.05–1.40, P = 0.009), elevated WBC (aOR = 1.15, 95% CI: 1.01–1.30, P = 0.035), and ART use (aOR = 1.63, 95% CI: 1.02–2.59, P = 0.041). BMI was no longer significantly associated with GDM risk in the adjusted model (aOR = 1.03, 95% CI: 0.96–1.11, P = 0.370). Collinearity diagnostics (VIF< 2) indicated no substantial multicollinearity among the predictors, (**Table 4**).

**Table 4:** Multivariable logistic regression analysis of independent risk factors for GDM in advanced maternal age primiparas.

Variables	Univariate analysis					Multivariate analysis					VIF
	β	S.E	Z	P	OR (95%CI)	β	S.E	Z	P	aOR (95%CI)	
ART	0.38	0.22	1.73	0.084	1.46 (0.95 - 2.25)	0.49	0.24	2.05	0.041	1.63 (1.02 - 2.59)	1.008
U-GLU	1.88	0.52	3.65	<0.001	6.56 (2.39 - 18.03)	2.07	0.55	3.73	<0.001	7.91 (2.67 - 23.46)	1.013
FBG	0.63	0.31	2.06	0.040	1.87 (1.03 - 3.41)	0.80	0.35	2.32	0.020	2.23 (1.13 - 4.38)	1.032
NEU	0.25	0.06	4.29	<0.001	1.29 (1.15 - 1.45)	0.19	0.07	2.61	0.009	1.21 (1.05 - 1.40)	1.590
WBC	0.23	0.05	4.35	<0.001	1.25 (1.13 - 1.39)	0.14	0.07	2.11	0.035	1.15 (1.01 - 1.30)	1.632
BMI	0.08	0.03	2.24	0.025	1.08 (1.01 - 1.16)	0.03	0.04	0.90	0.370	1.03 (0.96 - 1.11)	1.076

The multivariable logistic regression model included the following independent variables: ART, assisted reproductive technology; U-GLU, urinary glucose positivity; FBG, fasting blood glucose; NEU, neutrophil count; WBC, white blood cell; BMI, body mass index. Covariates were selected based on univariate analysis (P< 0.10) and clinical relevance. Collinearity was assessed via variance inflation factor (VIF), with all VIFs< 2, indicating absence of multicollinearity. The dependent variable was GDM status (yes/no). Adjusted odds ratios (aOR) with 95% confidence intervals (CI) are presented.

### Forest plot of risk factors for GDM in advanced maternal age nulliparous women

**Figure 2:**
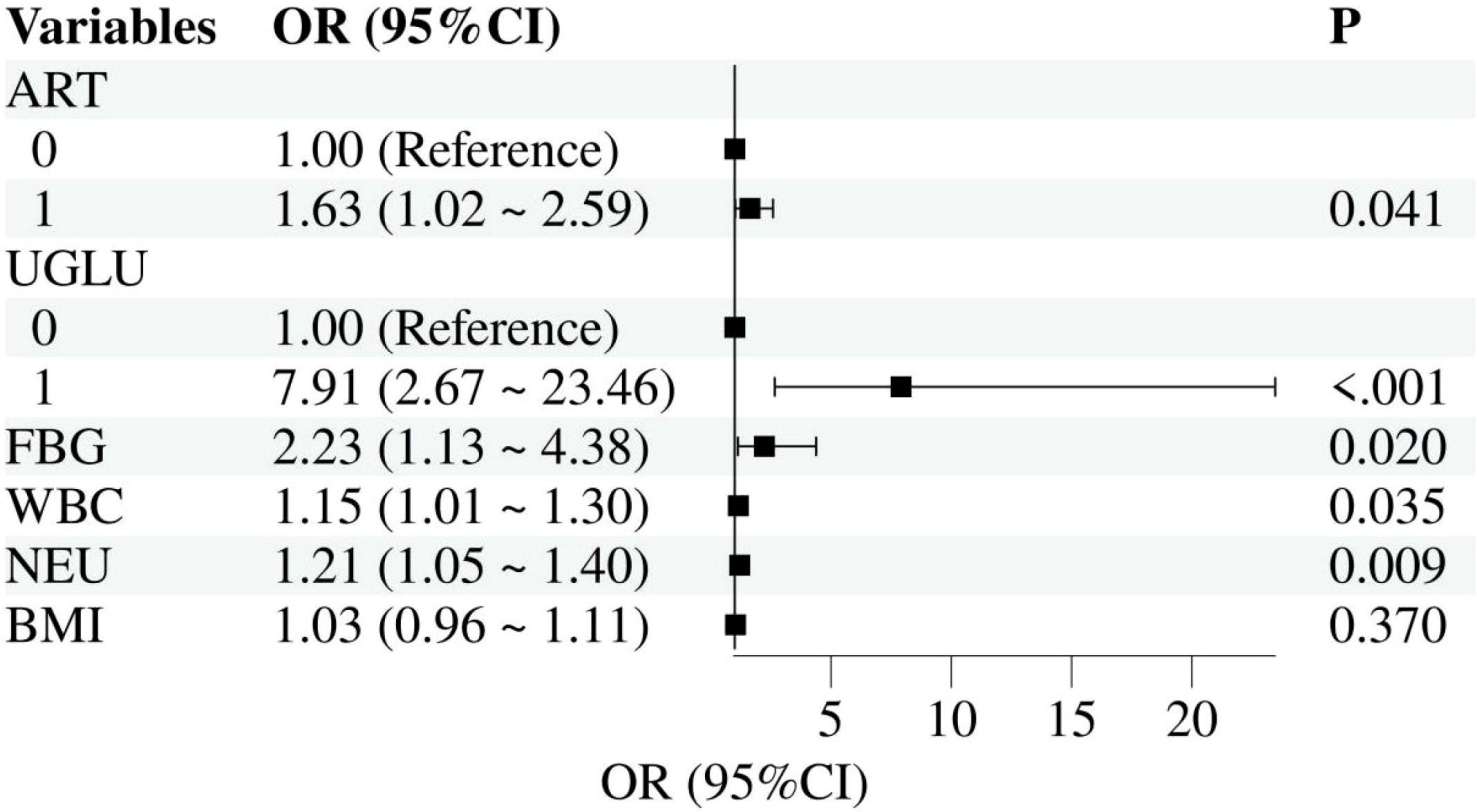
Forest plot of risk factors for GDM in advanced maternal age nulliparous women. ART, assisted reproductive technology; U-GLU, urinary glucose positivity; FBG, fasting blood glucose; NEU, neutrophil count; WBC, white blood cell; BMI, body mass index.

U-GLU: The aOR is 7.91 with a 95% CI of 2.67 to 23.46, far from 1, indicating a highly significant risk increase. NEU: The aOR is 1.21 with a 95% CI of 1.05 to 1.40, which does not include 1, representing a 21% increased risk. WBC: The aOR is 1.15 with a 95% CI of 1.01 to 1.30, which does not include 1, representing a 15% increased risk.ART and FBG: Both aORs are greater than 1 and their 95% CIs do not include 1 (ART: 1.02–2.59; FBG: 1.13–4.38), confirming a positive association with GDM. Overall, the forest plot illustrates a strong correlation between U-GLU,WBC, NEU and ART as risk factors for GDM in this population (**Figure 2**).

### Assessment of predictor importance using SHAP values

**Figure 3:**
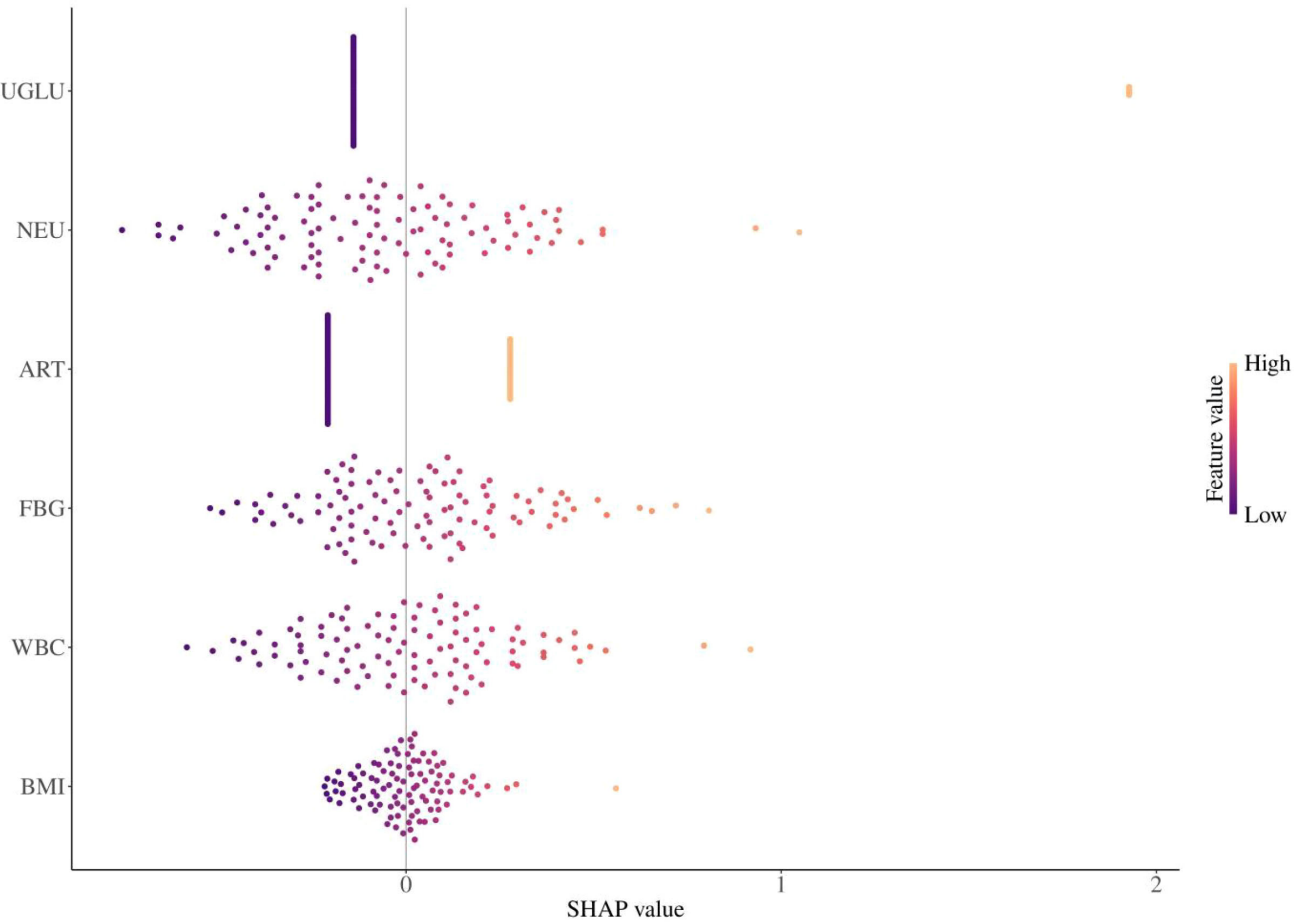
Assessment of predictor importance using SHAP values. Each point represents a single patient. The position on the x-axis shows the impact (SHAP value) of that variable on the model prediction for that patient (positive values push the prediction towards GDM, negative values towards non-GDM). The color represents the actual value of the variable for that patient (red = high, blue = low). The variables are ordered from top to bottom by their mean absolute SHAP value (overall importance).

SHAP analysis was performed to quantify the relative contribution of each independent risk factor to the model’s predictions. U-GLU positivity exhibited the highest mean absolute SHAP value, confirming its role as the most influential predictor, followed by NEU, ART, FBG, WBC, and BMI. Beyond confirming the logistic regression findings, SHAP provides complementary insights by visualizing the direction and magnitude of each predictor’s effect at the individual patient level. For example, high U-GLU values (red) consistently increased predicted GDM risk (positive SHAP values), while low U-GLU values (blue) decreased risk—a bidirectional impact not captured by a single odds ratio. This individual-level heterogeneity was also observed for continuous variables such as NEU and FBG, where higher values were associated with increased risk and lower values with decreased risk, consistent with expected biological relationships (**Figure 3**).

### Model performance

**Figure 4:**
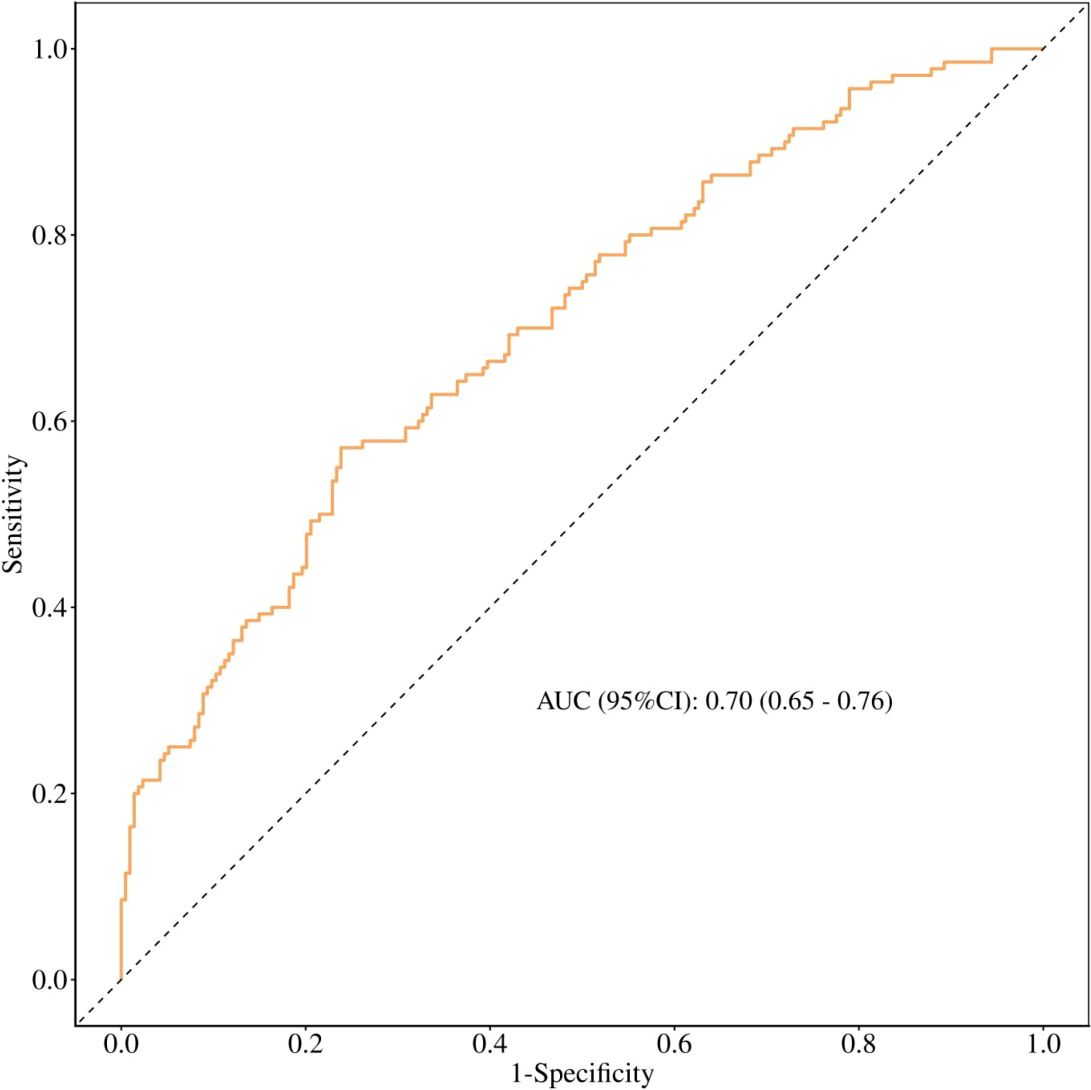
Receiver operating characteristic (ROC) curve for the multivariable logistic regression model predicting GDM.

The multivariable logistic regression model (including U-GLU, FBG, WBC, NEU, ART, and BMI) achieved an area under the receiver operating characteristic curve (AUC) of 0.70 (95% CI 0.65 - 0.76) (**Figure 4**).

**Figure 5:**
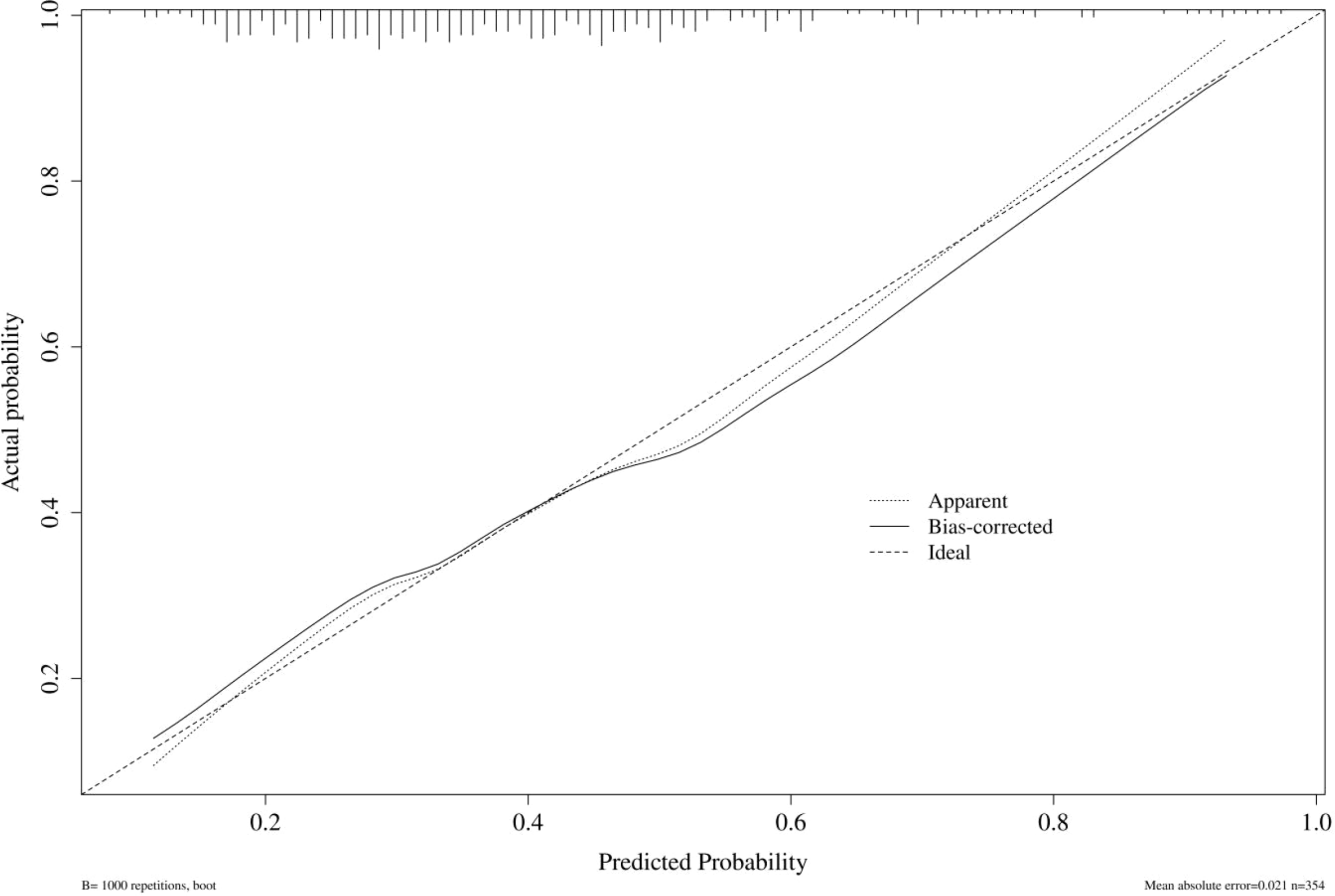
Calibration plot of predicted versus observed GDM risk.

The calibration plot showed good agreement between predicted and observed probabilities, and the Hosmer–Lemeshow test yielded a non-significant P-value of 0.704, indicating good calibration (**Figure 5**).

**Figure 6:**
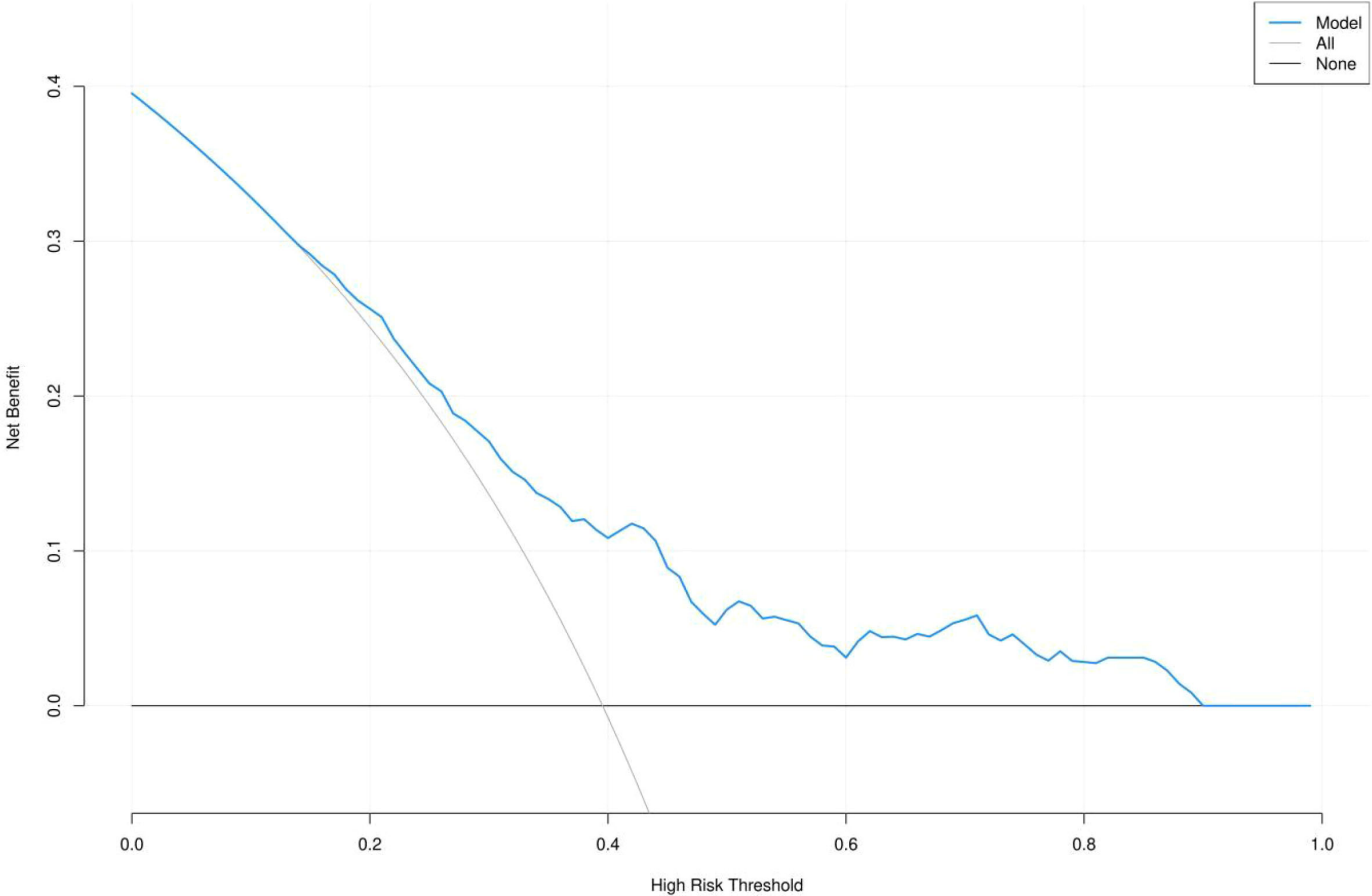
Decision curve analysis for the multivariable model.

At the optimal probability cutoff determined by the Youden index, the model had a sensitivity of 57.1%, specificity of 76.2%, positive predictive value (PPV) of 60.9%, and negative predictive value (NPV) of 73.1%. Decision curve analysis demonstrated that the model provided a positive net benefit across a range of clinically relevant threshold probabilities, with the net benefit curve remaining above both the “treat all” and “treat none” strategies for thresholds approximately between 0.2 and 0.8 (**Figure 6**).

## Discussion

This retrospective observational study identifies associations, not causal relationships, between early-pregnancy biomarkers and subsequent GDM in nulliparous women of advanced maternal age. The findings should be interpreted with caution, and causal inference cannot be drawn from this study design. This study analyzed retrospective data from 520 AMA nulliparous women and found no significant differences in baseline characteristics—including age, BMI, parity, and age at menarche—between the complete data set (n = 354) and the incomplete data set (n = 166) (P > 0.05). These findings confirm that missing data did not introduce selection bias, thereby providing a robust foundation for subsequent analyses. When comparing GDM and non-GDM groups, the GDM cohort demonstrated a significant dual metabolic and inflammatory burden. Metabolically, this was evidenced by elevated fasting blood glucose levels (4.71 vs. 4.63 mmol/L, P = 0.038) and a markedly higher prevalence of U-GLU positivity (13.57% vs. 2.34%, P< 0.001). From an inflammatory perspective, the GDM group showed significantly increased white blood cell counts (9.11 vs. 8.00×10^9^/L, P< 0.001) and NEU (6.50 vs. 5.63×10^9^/L, P< 0.001). Notably, U-GLU emerged as the strongest independent predictor of GDM in multivariate logistic regression analysis (OR = 7.91, 95% CI: 2.67–23.46), exceeding traditional risk factors such as BMI (P = 0.486) in predictive strength. Our findings corroborate existing evidence of heightened metabolic risk in advanced maternal age pregnancies (1, 5, 8) and suggest that impaired renal glucose threshold may represent an early warning sign for GDM. Furthermore, ART remained an independent risk factor (OR = 1.63, 95% CI: 1.02–2.59), highlighting its contributory role in GDM development among this population. These results offer novel insights into the complex pathophysiology of GDM in nulliparous women of advanced maternal age.

### ART as a risk factor for GDM

Our ART cohort, primarily consisting of IVF-ET cases, comprised 152 women, including 5 cases of IUI. Due to the limited sample size of IUI cases, this study pooled ART procedures as a composite variable to analyze their impact, thus primarily reflecting risk characteristics within the IVF-ET population. Multivariable logistic regression analysis revealed an adjusted odds ratio (OR) of 1.63 (95% CI: 1.02–2.59) for ART, indicating a statistically significant increase in GDM risk after controlling for confounding variables. This finding aligns with previous literature: Study (15) identified IVF as an independent risk factor for GDM, particularly among women with overweight status (BMI > 25 kg/m²). The same study further demonstrated that IVF is associated not only with increased GDM incidence but also with abnormal glycemic profiles (15). Another investigation (16), after adjustment for maternal age, BMI, and other confounders, reported a significant elevation in GDM risk in ART-conceived pregnancies (OR 1.30). Additional research indicates that ART is often correlated with advanced maternal age and nulliparity—known risk factors for GDM—potentially exerting a compounded effect on disease development (17).

Mechanistically, ART may elevate GDM risk via endocrine disruption and metabolic dysregulation. The ART process involves ovarian stimulation and hormonal regulation, which can impair insulin sensitivity and promote inflammatory pathways. For example, studies have shown that overweight women undergoing IVF have a higher propensity for GDM, suggesting a synergistic effect between ART-related metabolic perturbations and obesity, disrupting glucose homeostasis (15). Moreover, ART-associated alterations in maternal physiological states, such as elevated inflammatory markers, could contribute to GDM pathogenesis (18). These mechanistic insights explain why ART emerges as a significant risk factor in multivariable models, whereas its significance may be attenuated in univariate analyses that do not fully account for confounding variables like maternal age or obesity.

Furthermore, infertility indications that independently affect GDM risk (e.g., PCOS, endometriosis) were not available in this retrospective study, which may confound the observed association between ART and GDM. This should be considered when interpreting our findings.

### Inflammatory markers and the risk of GDM

Inflammation plays a central role in the pathogenesis of GDM, primarily through systemic inflammatory activation that induces insulin resistance and disturbances in glucose metabolism. Multiple studies have confirmed that inflammatory biomarkers such as elevated WBC and NEU counts reflect an underlying systemic inflammatory state. This state promotes the release of pro-inflammatory cytokines, including interleukin-6 (IL-6), which can directly impair insulin signaling pathways, leading to glucose uptake disturbances (19). Consistent with our analyses, the GDM group exhibited significantly higher NEU and WBC counts. NEU was independently associated with increased GDM risk (OR = 1.21). This effect aligns with observations from acute inflammatory states (e.g., severe infections), underscoring the pivotal role of systemic inflammation in metabolic dysregulation. Furthermore, this highlights ferritin as a key inflammatory biomarker involved in systemic inflammatory responses (20). Activation of maternal inflammatory pathways, such as NF-κB signaling, leads to the release of pro-inflammatory cytokines like tumor necrosis factor-alpha (TNF-α). These cytokines impair pancreatic β-cell function and adversely affect adipose tissue metabolism, thereby exacerbating insulin resistance and glucose intolerance (21), ultimately increasing GDM risk. Neutrophil counts in early pregnancy are considered sensitive predictors of GDM development, often outperforming other hematological parameters such as WBC count (22), which aligns with our finding of NEU serving as an independent predictive marker. These inflammatory biomarkers offer potential for early screening of GDM, especially when integrated with other risk factors, to improve predictive accuracy. Additionally, large-scale cohort retrospective and prospective studies have shown that elevated Hb levels in early pregnancy (defined as Hb ≥13.2 g/dL) are associated with increased GDM risk, possibly because higher Hb often reflects concurrent elevations in ferritin, a significant inflammatory marker (23, 24). Although the difference in Hb levels between groups was not statistically significant, the GDM group had higher mean Hb levels.

While we hypothesize that pro-inflammatory cytokines such as interleukin-6 (IL-6) and tumor necrosis factor-alpha (TNF-α) may mediate the link between neutrophilia and GDM pathogenesis, these specific mediators were not directly measured in this study. Our conclusions regarding systemic inflammation are therefore inferred from elevated neutrophil counts—a well-validated surrogate marker of innate immune activation—and supported by existing literature demonstrating downstream effects on insulin signaling pathways (17, 20). Future prospective studies should incorporate direct measurements of circulating cytokines to confirm these mechanistic links.

### Metabolic factors and the risk of GDM

FBG levels were significantly higher in the GDM group, and FBG was identified as an independent risk factor (OR = 2.23). U-GLU positivity was markedly more prevalent in the GDM group and emerged as the strongest predictor (OR = 7.91). Elevated FBG reflects early β-cell dysfunction and impaired insulin secretion; increases in FBG suggest compromised β-cell reserve, which is directly associated with GDM progression (25). Abnormal fasting glucose levels are early markers of insulin resistance (IR) and β-cell decompensation, with these effects being more pronounced in advanced maternal age due to age-related decline in β-cell function (26, 27). Chronic inflammatory states in advanced maternal age accelerate disruptions in insulin signaling pathways, such as IRS-1 phosphorylation inhibition, resulting in increased hepatic gluconeogenesis and fasting hyperglycemia (28, 29). The proportion of women with U-GLU positivity was markedly higher in the GDM group (13.57% vs. 2.34%). Multivariable regression analysis revealed that U-GLU positivity was strongly associated with GDM (OR = 7.91, 95% CI: 2.67–23.46), indicating U-GLU as a significant predictor. Higher U-GLU positivity correlates with lower renal glucose thresholds. During pregnancy, physiological glomerular hyperfiltration, coupled with hyperglycemia in GDM, exceeds the renal threshold for glucose reabsorption, leading to glycosuria (30, 31). Furthermore, hyperglycemia can induce oxidative stress and promote the release of inflammatory cytokines such as TNF-α and IL-6, which further impair renal SGLT transporter function (32, 33), creating a positive feedback loop of “hyperglycemia → renal damage → glycosuria.” Metabolic factors associated with hyperglycemia, such as U-GLU, play crucial roles particularly in insulin-requiring GDM, potentially through mechanisms involving decreased renal glucose thresholds and dysregulated glucagon secretion (34). Additionally, two Chinese studies have demonstrated that FBG in early pregnancy is an independent predictor of GDM onset (35, 36). The significant differences observed in metabolic markers like FBG and U-GLU in our cohort support the concept of metabolic heterogeneity in GDM. Given our focus on advanced maternal age and primiparous women, the age structure may have amplified the relative importance of metabolic factors. The Chinese guidelines for the diagnosis and management of gestational hyperglycemia (2022 edition) (13) recommend that all pregnant women undergo FBG testing at their first prenatal visit. Based on our findings, early pregnancy screening for FBG and U-GLU is essential for the early identification of GDM risk factors, facilitating timely and targeted interventions to improve maternal and fetal outcomes.

It is important to note that physiological changes during pregnancy—including glomerular hyperfiltration and increased renal plasma flow—can lower the renal threshold for glucose reabsorption, potentially contributing to glycosuria even in normoglycemic women. However, in the context of early hyperglycemia, as reflected by elevated FBG, the combination of reduced renal threshold and higher filtered load likely amplifies U-GLU excretion. Thus, U-GLU positivity in early pregnancy may reflect not only metabolic dysregulation but also altered renal handling of glucose under hormonal influence. Longitudinal assessment of estimated glomerular filtration rate (eGFR) and SGLT transporter function could further clarify this interaction in future studies.

### Model performance considerations

Although the primary aim of this study was to identify early-pregnancy risk factors rather than to develop a clinical prediction model, we evaluated the discriminative ability of the combined risk factors. The multivariable model achieved an AUC of 00.70 (95% CI 0.65 - 0.76 ), indicating acceptable discrimination. The calibration plot and Hosmer–Lemeshow test (P = 0.704) demonstrated good agreement between predicted and observed probabilities. Decision curve analysis further suggested that the model could provide clinical net benefit across a range of threshold probabilities. These findings support the potential utility of integrating these readily available biomarkers for early risk stratification, although prospective validation is needed before clinical implementation.

### Clinical implications

Based on these findings, we propose a potential tiered screening strategy for AMA nulliparous women. At the first prenatal visit (10–13 weeks), routine measurement of FBG, complete blood count (providing NEU), and urinalysis (U-GLU) would be performed. Women with U-GLU positivity, elevated FBG, or elevated NEU could be flagged as high-risk and receive early lifestyle counseling, while still undergoing the standard 24–28 week OGTT. This strategy leverages tests already routine in prenatal care, incurring minimal additional cost. Compared to FBG alone, adding U-GLU and NEU improved risk discrimination AUC = 0.70, with U-GLU positivity identifying a subset at markedly elevated risk (OR = 7.91). However, the true incremental benefit—in terms of improved outcomes and cost-effectiveness—requires prospective validation before clinical implementation.

### Role and limitations of BMI in GDM prediction

Although univariate analysis showed significantly higher BMI in the GDM group, BMI did not retain an independent association in the multivariable model. This suggests that BMI may influence GDM risk indirectly through interactions with other factors, such as FBG or NEU, rather than serving as a direct driving factor. These findings align with prior studies that identify obesity as a risk factor for GDM but emphasize its complex interplay with non-genetic factors such as inflammation and insulin resistance (1). The current results also correspond with previous research indicating that BMI’s impact on GDM may be context-dependent, especially in populations with relatively homogeneous BMI distributions. In particular, obesity combined with GDM can contribute to a “gestational metabolic syndrome” (37), but our multivariable model shows BMI does not independently contribute to GDM risk. This underscores the prior importance of metabolic and inflammatory biomarkers over BMI in this specific cohort. The limited discriminative power of BMI in this population may relate to the narrow age range (interquartile range: 35–38 years), which reduces variability, and suggests that BMI’s influence might be largely mediated through metabolic parameters such as FBG. Existing literature indicates that BMI’s role in GDM may vary according to demographic characteristics and population-specific factors (38, 39), and in our cohort, the relatively low median BMI (~22 kg/m²) may limit its predictive value for GDM in this particular subgroup. Overall, while BMI remains a relevant risk factor in broader populations, in high-risk older primiparous women, its independent predictive capacity may be overshadowed by more direct metabolic and inflammatory indicators.

Compared to traditional risk stratification models relying solely on BMI and family history, our proposed approach integrates easily accessible early-pregnancy biomarkers—namely U-GLU, NEU, WBC, FBG, and ART status—that collectively capture both metabolic and inflammatory pathways involved in GDM development. Notably, U-GLU emerged as the strongest and most consistent predictor, with the highest adjusted odds ratio (aOR=7.91) in the regression model and the highest mean absolute SHAP value in the complementary importance analysis. This underscores that simple point-of-care urinalysis, often overlooked in routine screening, may serve as a powerful tool for identifying high-risk individuals before overt hyperglycemia develops. Implementing a tiered screening strategy incorporating these markers could improve early detection rates and allow timely interventions.

### Limitations

Our study has several limitations. Its single-center, retrospective design inherently limits causal inference and may introduce unmeasured confounding. Although we adjusted for multiple confounders, residual confounding due to unmeasured factors such as family history of diabetes, polycystic ovary syndrome, infertility etiology, and prior impaired glucose tolerance cannot be ruled out. Additionally, complete-case analysis was used, and 166 women (32% of the initial cohort) were excluded because they did not undergo early prenatal care or laboratory assessments at our hospital during the first trimester, and thus their complete first-trimester data were unavailable. Although baseline characteristics were comparable between included and excluded women, this high exclusion rate may still introduce selection bias if missingness was not completely at random, and may reduce statistical power. Due to the small sample size of the IUI subgroup (n=5), ART was analyzed as a composite variable; therefore, our findings regarding ART primarily reflect the risk associated with IVF-ET and may not be generalizable to IUI. Furthermore, infertility indications that independently affect GDM risk (e.g., PCOS, endometriosis) were not available, potentially confounding the observed ART–GDM association. Urinary glucose was assessed by a single semi-quantitative dipstick test on random spot urine samples, which may be influenced by recent dietary carbohydrate intake and does not provide quantitative measurement. Although automated reading reduced inter-observer variability, physiological glycosuria due to reduced renal glucose threshold in pregnancy may have led to false positives, and we cannot definitively distinguish pathological from physiological glycosuria. Repeated testing or confirmatory quantitative urine glucose measurement would strengthen future studies. Inflammatory markers were limited to WBC and NEU counts; direct measurements of pro-inflammatory cytokines (e.g., IL-6, TNF-α) or other mediators (e.g., hs-CRP) were not performed. Similarly, we lacked data on HbA1c, insulin resistance indices (e.g., HOMA-IR), and longitudinal renal function (e.g., eGFR), which could provide deeper mechanistic insights. The study population comprised exclusively Chinese nulliparous women with advanced maternal age and relatively low median BMI (22.2 kg/m²); therefore, the findings may not be directly generalizable to other ethnic groups (e.g., Western populations with higher baseline GDM risk), women with obesity, or multiparous women. Finally, although we evaluated model performance (AUC = 0.70, sensitivity 57.1%, specificity 76.2%), these metrics are derived from the same dataset used to develop the model and may be optimistic; internal or external validation in independent cohorts is needed to confirm the model’s robustness.

### Future directions

Future research should aim to expand sample sizes and adopt multicenter prospective cohort designs. Studies should incorporate dynamic inflammation and metabolic markers—such as CRP, OGTT data—to more thoroughly elucidate the pathogenesis of GDM and improve early predictive models.

## Conclusions

In this single-center retrospective cohort of AMA nulliparous women, early-pregnancy urinary glucose positivity, elevated fasting blood glucose, neutrophilia, leukocytosis, and the use of assisted reproductive technology were independently associated with an increased risk of gestational diabetes mellitus. Among these, urinary glucose positivity demonstrated the strongest association. These findings suggest that a tiered screening strategy incorporating these readily available metabolic and inflammatory biomarkers between 10 and 13 weeks of gestation could potentially enhance early risk stratification in this high-risk population. The clinical utility and predictive performance of this approach require prospective validation in larger, multicenter studies.

## Data Availability

The original contributions presented in the study are included in the article/supplementary material. Further inquiries can be directed to the corresponding authors.
